# Implications of fALS Mutations on Sod1 Function and Oligomerization in Cell Models

**DOI:** 10.1007/s12035-017-0755-4

**Published:** 2017-09-07

**Authors:** Aline A. Brasil, Rayne S. S. Magalhães, Mariana D. C. De Carvalho, Isabel Paiva, Ellen Gerhardt, Marcos D. Pereira, Tiago F. Outeiro, Elis C. A. Eleutherio

**Affiliations:** 1 Department of Experimental Neurodegeneration, Center for Nanoscale Microscopy and Molecular Physiology of the Brain, Center for Biostructural Imaging of Neurodegeneration, University Medical Center Göttingen, 37073 Göttingen, Germany; 2 Departamento de Química, Instituto de Química, Centro de Tecnologia, Cidade Universitária, Universidade Federal do Rio de Janeir, Av. Athos da Silveira Ramos, n 149, Bloco A - sala 547, Rio de Janeiro, RJ 21941-909 Brazil; 3 Max Planck Institute for Experimental Medicine, Göttingen, Germany

**Keywords:** Sod1, fALS, Neurodegeneration, Oxidative stress

## Abstract

Among the familial forms of amyotrophic lateral sclerosis (fALS), 20% are associated with the Cu,Zn-superoxide dismutase (Sod1). fALS is characterized by the accumulation of aggregated proteins and the increase in oxidative stress markers. Here, we used the non-invasive bimolecular fluorescence complementation (BiFC) assay in human H4 cells to investigate the kinetics of aggregation and subcellular localization of Sod1 mutants. We also studied the effect of the different Sod1 mutants to respond against oxidative stress by following the levels of reactive oxygen species (ROS) after treatment with hydrogen peroxide. Our results showed that only 30% of cells transfected with A4VSod1 showed no inclusions while for the other Sod1 mutants tested (L38V, G93A and G93C), this percentage was at least 70%. In addition, we found that 10% of cells transfected with A4VSod1 displayed more than five inclusions per cell and that A4V and G93A Sod1 formed inclusions more rapidly than L38V and G93C Sod1. Expression of WTSod1 significantly decreased the intracellular oxidation levels in comparison with expression of fALS Sod1 mutants, suggesting the mutations induce a functional impairment. All fALS mutations impaired nuclear localization of Sod1, which is important for maintaining genomic stability. Consistently, expression of WTSod1, but not of fALS Sod1 mutants, reduced DNA damage, as measured by the comet assay. Altogether, our study sheds light into the effects of fALS Sod1 mutations on inclusion formation, dynamics, and localization as well as on antioxidant response, opening novel avenues for investigating the role of fALS Sod1 mutations in pathogenesis.

## Introduction

Amyotrophic lateral sclerosis (ALS) is an age-associated neurodegenerative disease characterized by progressive muscle paralysis and death [1]. Approximately 10% of ALS cases are associated with autosomal dominant mutations (fALS) [2]; among them, 20% are caused by Cu, Zn-superoxide dismutase (Sod1) mutations [3]. Sod1 is an abundant and important protein in eukaryotic cells. Its physiological function is mainly related to the reduction of oxidative stress by converting superoxide anions to hydrogen peroxide, which is in turn broken down to water and oxygen by peroxidases [4]. Recently, Sod1 was reported to translocate into the nucleus of yeast cells in response to oxidative stress, where it regulates the expression of antioxidant and repair genes [5].

More than 150 individual mutations have been identified in human Sod1 (hSod1), affecting in different ways the onset and the prognosis of fALS [6, 7]. Nevertheless, despite intense studies, the molecular mechanisms connecting mutation hSod1 to fALS etiology and pathology are still elusive. The toxicity of fALS hSod1 has been related to structural instability [8], misfolding, aberrant enzymatic activity (lost and gain of function) [9], and disturbance of redox homeostasis [10] as well as to the presence of Sod1-containing aggregates in juxtanuclear quality control compartment (JUNQ) [11]. In this context, while transgenic mice expressing the fALS-associated hSod1 mutants G93A and A4V exhibit ALS-like phenotypes, animal knockouts for endogenous Sod1 do not [9, 12].

In vitro studies have shown that metal coordination and disulfide bond formation are relevant for Sod1 structural stability [13–15], suggesting that an unfolded Sod1 conformation is responsible for the increased aggregation propensity. In addition, it has been shown that aggregation propensity of recombinant Sod1 does not always predict aggregation in a cellular context. It is not surprising due to obvious differences in protein concentration and surrounding environment between in vitro and in cell [16]. Therefore, in this study, we set out to assess the contribution of fALS hSod1 (A4V, L38V, G93A and G93C) towards protein aggregation, oxidative stress, and DNA damage. Using the bimolecular fluorescence complementation (BiFC) assay, which allows to observe protein interactions in vivo, we found that these Sod1 mutations induce defective dimerization and, consequently, affect protein aggregation [17]. Importantly, we investigated, for the first time, the dynamics of fALS Sod1 aggregation in living cells and the subcellular partitioning of Sod1 inclusions in respect to the JUNQ compartment. We found that the fALS-associated hSod1 mutants tested are unable to overcome oxidative stress and DNA damage.

Altogether, our results clearly define a common mechanism of action of mutant Sod1, resulting in cytotoxicity, impaired function, aggregation, and in alterations in intracellular distribution, opening novel territory for the identification of targets for therapeutic intervention in ALS.

## Material and Methods

### Bimolecular Fluorescence Complementation Plasmids

The cDNA sequence of human WT (wild-type) *SOD1* and the mutants A4V, L38V, G93A, and G93C were subcloned from the yeast plasmid YEp351 [18, 19] into the Venus-BiFC plasmids previously described [20]. In particular, we used a larger N-terminal fragment of Venus (VN), corresponding to amino acids 1–158, and a smaller C-terminal fragment (VC), corresponding to amino acids 159–239. Human *SOD1* cDNA (WT, A4V, L38V, G93A and G93C) was cloned to the 3′-end of the VN-fragment (VN-*SOD1*) and upstream of the VC-fragment (*SOD1*-VC) by PCR, using specific primers including restriction enzyme sites AflII at the 5′ and XhoI at the 3′-end. The primers used were as follows:

- VN-SOD1 (WT, L38V, G93A, G93C)
  - Forward: 5′-GGGCTTAAGATGGCGACGAAGGCCGTG -3′
  - Reverse: 5′-CCCCTCGAGTTATTGGGCGATCCCAATTACACC -3′
- SOD1-VC (WT, L38V, G93A, G93C)
  - Forward: 5′-GGGCTTAAGATGGCGACGAAGGCCGTG -3′
  - Reverse: 5′-CCCCTCGAGTTGGGCGATCCCAATTACACCACAAG -3′
- VN-SOD1 (A4V)
  - Forward: 5′-GGGCTTAAGATGGCGACGAAGGTCGTGTGCG -3′
  - Reverse: 5′-CCCCTCGAGTTATTGGGCGATCCCAATTACACC -3′
- SOD1-VC (A4V)
  - Forward: 5′-GGGCTTAAGATGGCGACGAAGGTCGTGTGCG -3′
  - Reverse: 5′-CCCCTCGAGTTGGGCGATCCCAATTACACCACAAG -3′

PCR fragments were restriction digested and cloned into alpha-synuclein BiFC constructs by replacing the alpha-synuclein insert [20]. All constructs were verified by DNA sequencing.

### Cell Culture and Transfections

Human neuroglioma cells (H4) were cultured in Dulbecco’s Modified Eagle Medium (DMEM, Life Technologies-Invitrogen, CA, USA), supplemented with 10% (*v*/*v*) fetal bovine serum (FBS) gold and 1% (*v*/*v*) penicillin-streptomycin, at 37 °C, and 5% CO_2_ humidified atmosphere. Transfections were performed by calcium phosphate using equal amounts of plasmids encoding the wild-type (WT) or mutant (A4V, L38V, G93A and G93C) hSod1 fused to Venus BiFC system and the JUNQ substrate (mCherry-VHL). To improve the visualization of VHL-mCherry proteins into JUNQ compartments, 48 h transfected H4 cells were incubated with proteasome inhibitor MG132 (10 μM) for 7 h.

### Fluorescence Microscopy

Forty-eight hours after transfection, H4 cells were washed with Dulbecco’s phosphate-buffered saline (DPBS) and fixed with 4% paraformaldehyde (PFA) for 10 min at room temperature (RT). Followed by three washing steps with DPBS, cells were stained with Hoechst 33258 (Life Technologies-Invitrogen, Carlsbad, CA, USA) (1∶5000 in DPBS) for 5 min and maintained in DPBS for fluorescence microscopy. Fluorescence images were acquired with a Leica DMI 6000B microscope (Leica, Germany), with a ×40 objective. Scale bars were calculated by using ImageJ software and were included in the figure legends together with the actual magnification.

### Quantification of Nuclear and Cytoplasmic Fluorescence Intensities

Nuclear and cytoplasmic fluorescence intensities were quantified using ImageJ software (http://rsbweb.nih.gov/ij/). Using the freehand tool, the nucleus and cytosol were selected and the respective intensities were measured. The results reflect the counting of at least 50 cells per condition.

### Quantification of hSod1 Inclusions

Transfected cells were detected and scored based on the hSod1 inclusions pattern and classified into three groups: cells without inclusions, five or less inclusions (≤ 5 inclusions), and more than five inclusions (≥ 5 inclusions). Results reflect the counting of at least 50 cells per condition.

### Immunocytochemistry

Forty-eight hours after transfection, cells were fixed on coverslips with 4% (*v*/*v*) PFA, for 15 min at RT. After washing with ×1 PBS, cells were permeabilized with 0.1% (*v*/*v*) Triton/PBS, for 15 min at RT. After blocking with 3% (*w*/*v*) bovine serum albumin (BSA)/PBS for 1 h at RT, cells were incubated for 2 h with primary antibody anti-G3BP 1:200 (BD Transduction Laboratories, kind gift of Prof. Flaviano Giorgini, University of Leicester) diluted in blocking solution. Cells were washed with ×1 PBS before incubation with secondary Alexa Fluor antibody (mouse, 555, Life Technologies), prepared at 1:1000 in blocking solution, for 1 h at RT. Before mounting the coverslips with Mowiol (Calbiochem, Germany), nuclei were stained with 4′,6-diamidino-2-phenylindole (DAPI, Roth, Germany). Immunofluorescence images were acquired with Leica DMI 6000B microscope (Leica, Germany), using ×63 magnification objective.

### Live Cell Imaging

Images of H4 cells expressing BiFC-tagged hSod1 were recorded by using the Olympus IX81-ZDC microscope system (Olympus, Germany). Cells were maintained in DMEM, (Life Technologies-Invitrogen, CA, USA), supplemented with 10% (*v*/*v*) FBS and 1% (*v*/*v*) penicillin-streptomycin at 5% atmospheric CO_2_, with the incubation unit set to a temperature of 37 °C. Live cells were imaged every 30 min over a time course of 20 h overnight.

### Immunoblotting

Forty-eight hours post-transfection cells were washed with room temperature DPBS and then harvested. H4 cells were lysed with radioimmunoprecipitation assay (RIPA) lysis buffer (50 mM Tris pH 8.0, 0.15 M NaCl, 0.1% (*w*/*v*) SDS, 1% NP40 (*v*/*v*), 0.5% (*w*/*v*) Na-deoxycholate), 2 mM EDTA, and a protease inhibitor cocktail (one tablet/10 mL) (Roche Diagnostics, Mannheim, Germany). Protein concentration was determined using the Bradford assay (BioRad Laboratories, Hercules, CA, USA), and the gels were loaded with 30 μg protein after denaturation for 5 min at 95 °C in a protein sample buffer (125 mM of 1 M Tris HCl pH 6.8, 4% (*w*/*v*) SDS, 0.5% (*w*/*v*) bromophenol blue, 4 mM EDTA 20% (*v*/*v*) glycerol 10% (*v*/*v*) β-mercaptoethanol). Samples were separated on 15% (*w*/*v*) SDS-polyacrylamide gels (SDS-PAGE) with a constant voltage of 120 V using Tris-Glycine SDS 0.5% (*w*/*v*) running buffer (250 mM Tris, 200 mM Glycine, 1% (*w*/*v*) SDS, pH 8.3) for 75 min. Protein transference to PVDF membrane was carried out by using Trans-Blot^®^ Turbo™ Transfer System (Biorad, Hercules, CA, USA) during 30 min with constant current at 0.3 A. The membranes were blocked with 4% (*w*/*v*) BSA (Sigma-Aldrich, St. Louis, MO, USA) in ×1 TBS-Tween (50 mM Tris, 150 mM NaCl, 0.05% (*v*/*v*) Tween, pH 7.5) for 60 min at RT. The membranes were further incubated with primary antibody 1:2000 anti-hSod1 (sc-8637 Santa Cruz Biotechnology, INC) and mouse 1:5000 anti-γ-tubulin (T5326, produced by Sigma-Aldrich, St. Louis, MO, USA) in 4% (*w*/*v*) BSA/TBS-Tween overnight at 4 °C. After washing three times in TBS-Tween for 5 min, the membranes were incubated for 1 h with anti-mouse IgG and anti-goat IgG horseradish peroxidase labeled secondary antibody (GE Healthcare, Bucks, UK) at 1∶6000 in 4% (*w*/*v*) BSA/TBS-Tween. Detection was carried out using luminol reagent and peroxide solution (Millipore, Billerica, MA, USA) and applied to the membrane 1 min before scanning with in Fusion FX (Vilber Lourmat, Collégien, France). The band intensity was estimated using the ImageJ software (NIH, Bethesda, MD, USA) and normalized against γ-tubulin.

### Detection of DNA Damage

The comet assay, a single cell gel electrophoresis-based method, was used to the detect DNA single- and double-strand breaks, according to the protocol previously described in [21]. Agarose coated slides were prepared by dipping the slides into a 1% (*w*/*v*) low-gelling temperature agarose (PeqLab) and allowed to air-dry. Approximately 5 × 10^4^ cells/mL were harvested in DPBS, mixed with 1% agarose and placed on a precoated slide. After agarose has gelled, alkaline lysis was performed by submerging the slides in alkaline buffer (1.2 M NaCl, 100 mM Na_2_EDTA, 0.1% (*w*/*v*) sodium lauryl sarcosinate, 0.26 M NaOH, pH > 13) and stored overnight at 4 °C. After overnight lysis, the alkaline rinse solution (0.03 M NaOH, 2 mM Na_2_EDTA, pH = 12.3) was used to wash the slides three times and to conduct the electrophoresis for 25 min at a constant voltage of 0.6 V/cm. Slides were neutralized with distilled water and stained with 2.5 μg/mL of propidium iodide for 20 min at RT. DNA damage quantification was carried out by examining at least 50 comet images from each slide. The images were acquired with an epifluorescence microscope (Leica DMI 6000B microscope, Leica, Germany). CometScore software (TriTek Corp) was used to determine the tail moment (product of the tail length and the percentage of total DNA in the tail) of each individual comet image.

### Analysis of Intracellular Oxidation

The generation of intracellular reactive oxygen species (ROS) was assessed in H4 cells expressing BiFC-tagged hSod1 or untagged hSod1 (WT and mutants). Briefly, the cells were seeded in 48-well plates and washed once with DPBS. For ROS production measurement, cells were incubated with 25 μM 2′,7′-dichlorofluorescin diacetate (DCFDA, Sigma) at 37 °C for 30 min. Following the incubation period, two washing steps with DPBS were performed to remove excess probe and fluorescence intensity (excitation 485 nM; emission 535 nM) was measured using the microplate reader Infinite M2000 PRO, Tecan. After three basal measurements, cells were challenged with 5% (*v*/*v*) H_2_O_2._ The fluorescence values were recorded up to 45 min and normalized to those obtained from non-H_2_O_2_-induced cells.

### Statistical Analyses

Data were analyzed using Graph Pad Prism 5 (San Diego, California, USA) software and were expressed as the mean ± SD of at least three replicates. Statistical differences from WT Sod1 were calculated using one-way ANOVA and two-way ANOVA with Bonferroni correction. Significance was assessed for, where an asterisk corresponds to *p* < 0.05, double asterisks corresponds to *p* < 0.01, and triple asterisks corresponds to *p* < 0.001.

## Results

### fALS Mutations on hSod1 Promote Oligomerization and Impair Nuclear Localization

**Fig. 1:**
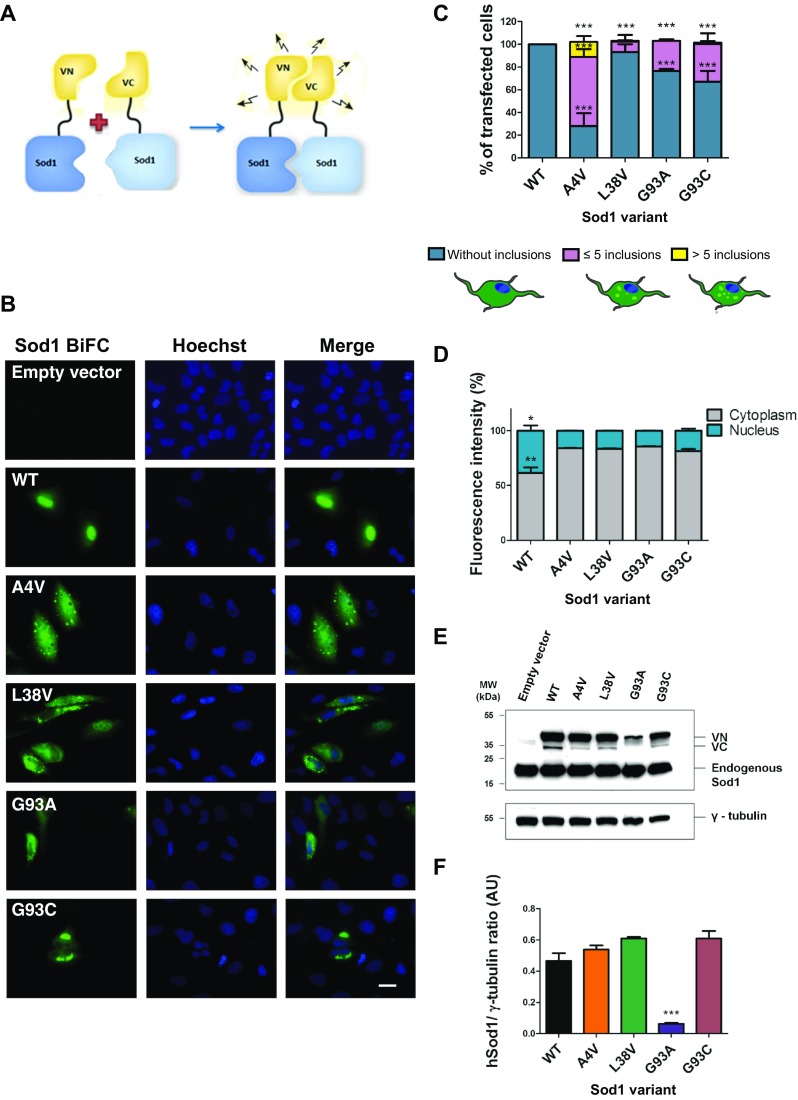
Sod1 mutations affect oligomerization subcellular distribution of inclusions. **a** Schematic representation of the bimolecular fluorescence complementation assay (BiFC). Sod1 fused to either the N- (VN-Sod1) or C-terminal (Sod1-VC) fragments of Venus. **b** Representative pictures of cells expressing fALS hSod1 mutants. H4 cells expressing WT or mutant hSod1 (A4V, L38V, G93A and G93C) VN-Sod1 and Sod1-VC constructs were analyzed by fluorescence microscopy. Scale bar 10 μm. Magnification ×1000. **c** Quantification of the number of inclusion per cell. At least 50 cells were counted per condition and classified in three different groups: blue, purple, and yellow bars represent the percentage of cells without any inclusion, with five or less inclusions and cells with more than five inclusions, respectively. Data was combined from at least three independent experiments. One-way ANOVA, with Bonferroni correction, was used for statistical analysis to compare differences between WT vs mutant cells (*** *p* < 0.001). **d** Subcellular localization of BiFC-hSod1. BiFC fluorescence intensity was quantified using ImageJ. For each condition, 50 cells were analyzed. **e** Representative immunoblot confirming expression of VN-Sod1 and Sod1-VC fragments. **f** Quantification of the immunoblots. Data is expressed as mean ± SD of at least three replicates. One-way ANOVA, with Bonferroni correction, was used for statistical analysis with significance level of *p* < 0.001 represented by three asterisks

In order to assess the effect of hSod1 mutations on oligomerization and subcellular localization, we used the BiFC-based assay which involves the fusion of Sod1 with non-fluorescent fragments of the Venus protein [22]. This assay is based on the reconstitution of a functional Venus fluorescent protein upon dimerization of hSod1, enabling the direct visualization of the formation of hSod1 oligomers and inclusions in living cells (Fig.1a). WT hSod1 formed only dimers/oligomers in H4 cells, but not inclusions, unlike all other variants tested (Fig. 1b, c). The inclusions formed by the hSod1 mutants tested exhibited different sizes and shapes (Fig. 1b). In addition, the number of inclusion per cell was also variable (Fig. 1c). For hSod1 A4V, 80% of the cells displayed small round inclusions. For the other mutants, around 30% of the cells displayed larger and elongated inclusions. A4V and L38V inclusions were found scattered over the cytoplasm, and G93A and G93C formed a larger number of inclusions per cell.

In addition, we also found that all fALS hSod1 mutations reduced the nuclear localization of Sod1 to half of that observed with WT hSod1 (Fig. 1b, d). The levels of A4V, G93C, and L38V hSod1 variants were identical, except those of G93A, suggesting this mutant may be more unstable (Fig. 1e, f).

**Fig. 2:**
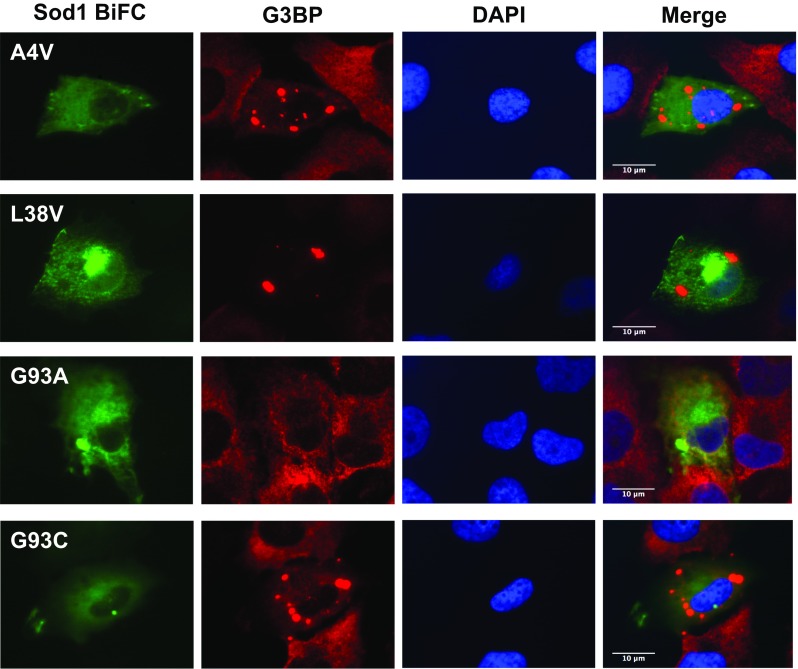
fALS mutant Sod1 inclusions do not colocalize with SGs. Inclusions of Sod1 were visualized by expression of BiFC-tagged Sod1 (green), and SGs were labeled by immunostaining with an antibody against G3BP (red). Nuclei were visualized with DAPI staining. Scale bar 10 μm. Magnification ×1000

Next, we asked whether the inclusions formed by Sod1 were localized in stress granules (SG), and costained cells with an antibody against G3BP, an established marker of SGs [23, 24]. We found that none of the inclusions formed by the hSod1 mutants tested in this study colocalized with G3BP (Fig. 2).

**Fig. 3:**
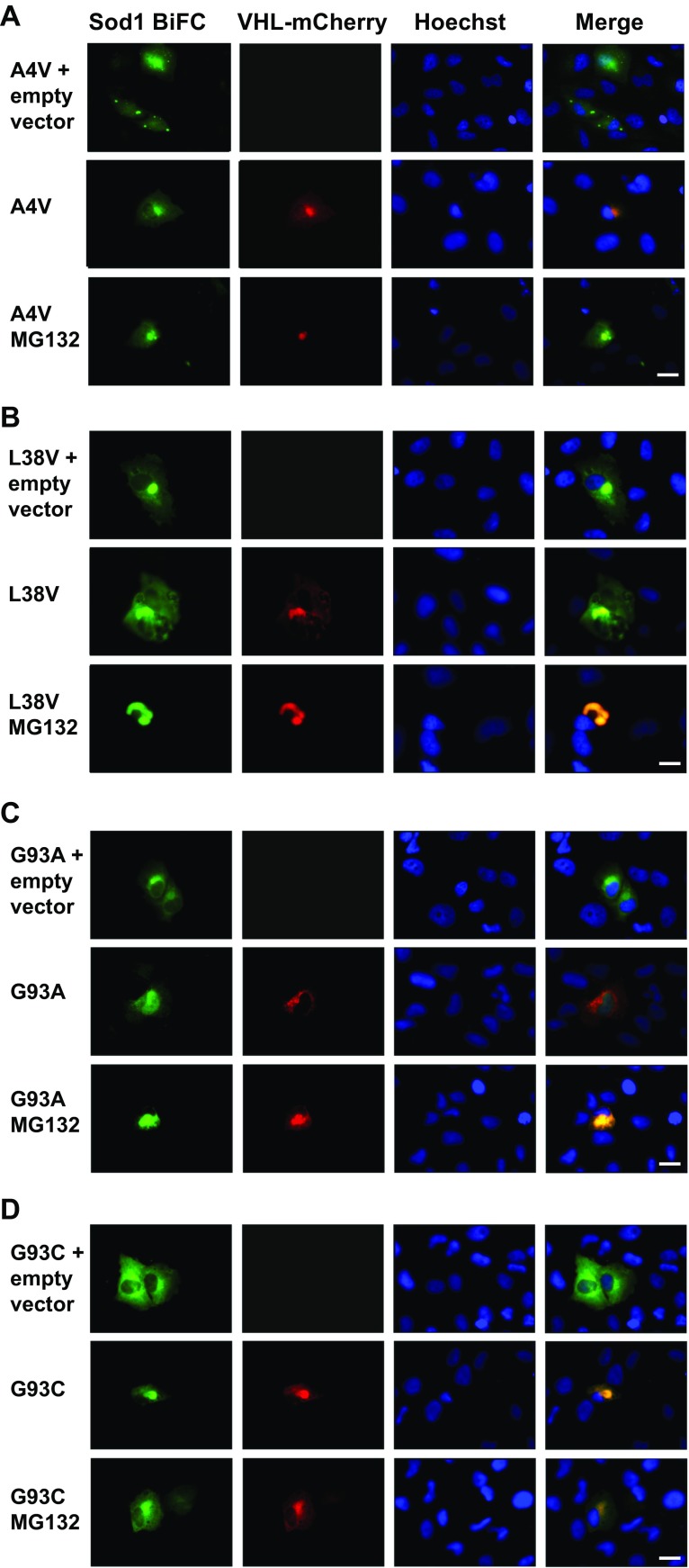
hSod1 mutants are preferentially directed to the JUNQ compartment in H4 cells. Colocalization of the hSod1 mutants with the VHL protein. As a control, H4 cells were transfected with pcDNA vector. Inclusions were visualized by expression of BiFC-tagged Sod1 (green) and JUNQ was observed by expression of VHL-mCherry (red), nuclei in all experiments were visualized with Hoechst staining. Representative pictures of BiFC. hSod1 A4V (**a**), L38V (**b**), G93A (**c**), and G93C (**d**) colocalizing with JUNQ. The inclusions formed by BiFC-tagged hSod1 A4V, L38V, and G93C are clearly visualized when proteasome is functional, whereas G93A inclusions are more observed into JUNQ after proteasome inhibition with MG132. Scale bar 20 μm. Magnification ×500

In order to investigate the site of accumulation of hSod1 in H4 cells, we monitored the localization of the inclusions using von Hippel-Lindau (VHL) fused to mCherry as a marker for the JUNQ compartment. VHL is a heterologous protein that misfolds and forms soluble aggregates that are targeted to the JUNQ compartment, where they colocalize with the quality control machinery [25]. To improve the visualization of VHL-mCherry proteins into JUNQ, H4 cells were incubated with the proteasome inhibitor MG132 (10 μM) for 7 h, 48 h after transfection. The inhibition of the proteasome did not change the visualization of the hSod1 variants into JUNQ, suggesting that the levels of fALS inclusions were sufficiently high under normal conditions (Fig. 3).

**Fig. 4:**
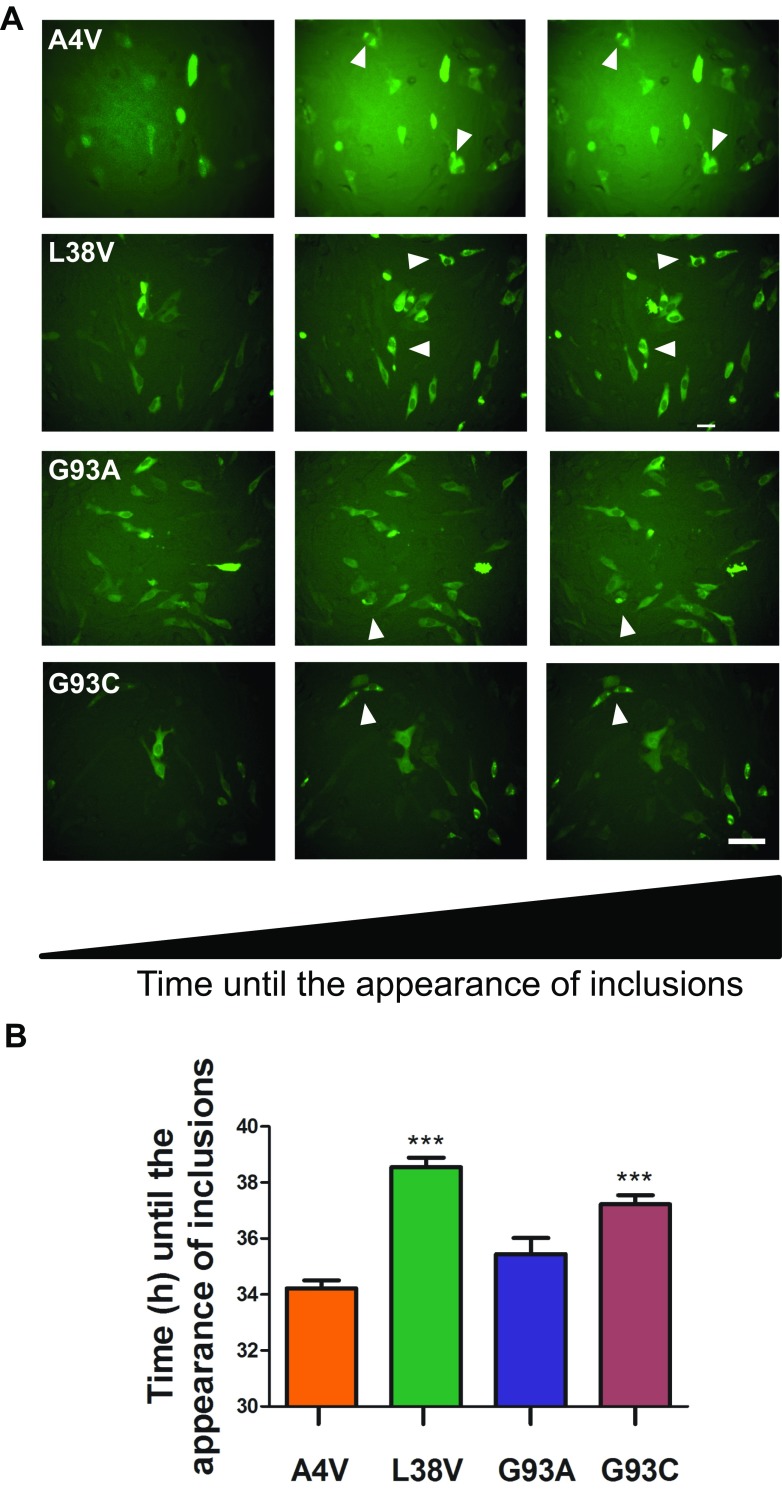
Live-cell imaging of cells expressing hSod1 BiFC constructs. **a** Representative frames of a time course showing inclusions in hSod1 BiFC expressing cells. Live-cell imaging was initiated 24 h post-transfection, and the pictures were taken every 30 min for 20 h. Scale bar 40 μm. Magnification ×250. **b** Chronological and comparative analyses of the dynamics of inclusions formation. Data is expressed as mean ± SD of at least three replicates. One-way ANOVA, with Bonferroni correction, was used for statistical analysis with significance level of *** *p* < 0.001, comparing hSod1A4V with L38V, G93C, and G93C hSod1 mutations

To investigate the dynamics of the formation of Sod1 inclusions in vivo, live-cell imaging was performed during 20 h post-transfection. The time of appearance of the first inclusions was recorded and compared among the variants of hSod1 tested. Upon careful analysis of the data, we verified that A4V and G93A hSod1 inclusions appeared about 3 h earlier than L38V and G93C hSod1 inclusions (Fig. 4) and persisted in the cells for more than 5 h. This clearly suggests a distinct aggregation dynamics between A4V/G93A and L38V/G93C fALS mutations.

### fALS hSod1 Mutants Do Not Efficiently Protect against ROS and DNA Damage

**Fig. 5:**
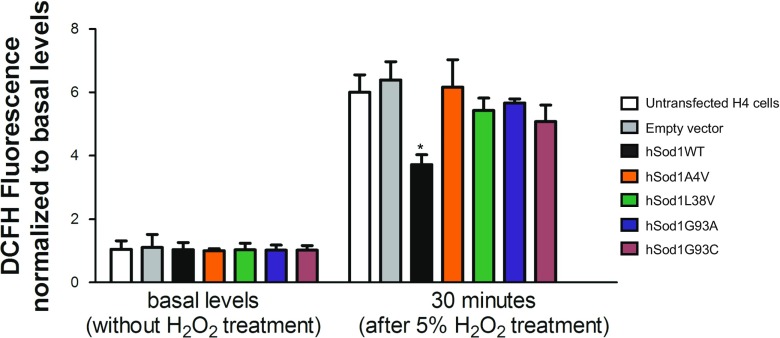
FALS mutants do not protect cells against ROS production. Intracellular ROS measurements were conducted in transfected H4 cells expressing the BiFC-tagged hSod1 by using the oxidant-sensing probe 2′,7′-dichlorofluorescein (DCFH). Thirty minutes after treatment with 5% H_2_O_2_, total cellular levels of ROS were significantly lower in H4 cells expressing BiFC-tagged WT Sod1 in comparison to fALS mutations A4V, L38V, G93A, and G93C levels. At basal levels, no differences in intracellular ROS between WT, and the four Sod1 mutations were observed. Data is expressed as mean ± SD of at least three replicates. Two-way ANOVA, with Bonferroni correction, was used for statistical analysis with significance level of * *p* < 0.05, comparing WT with Sod1 mutants

**Fig. 6:**
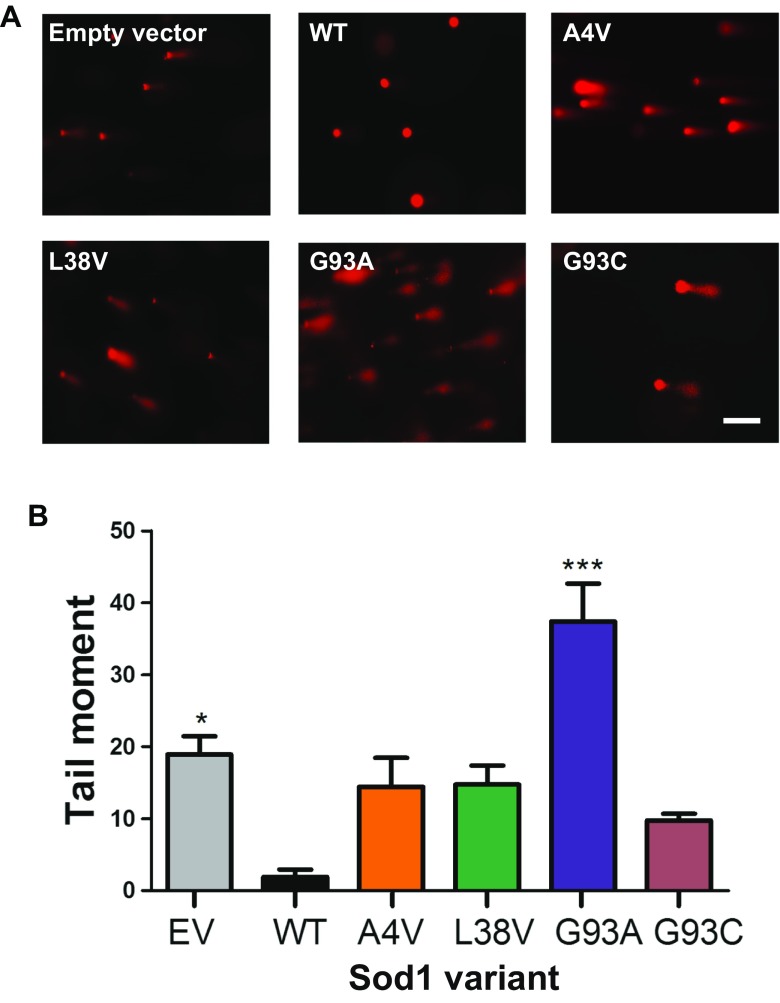
The G93A hSod1 mutation increases DNA damage in H4 cells. **a** Representative images of comet assay. Prestained comets with propidium iodide (PI) were imaged using fluorescence microscopy and tail moments were calculated using the Comet Score software. Scale bar: 40 μm. Magnification: 250X. **b** Levels of DNA damage in H4 cells expressing BiFC-tagged Sod1 WT and mutants. Quantification of double- and single-strand DNA breaks was made using alkaline comet assay method. BiFC-tagged hSod1expressing cells as well as control cells transfected with empty vector were all examined using the overnight alkaline method to analyze DNA damage. Data is expressed as mean ± SD of at least three replicates. One-way ANOVA, with Bonferroni correction, was used for statistical analysis between WT hSod1 and mutants hSod1 cells with significance level of an asterisk, *** *p* < 0.05

In order to assess whether BiFC-tagged Sod1 retains function, we measured intracellular ROS levels using the oxidative stress sensitive 2′,7′-dichlorofluorescein (DCFH) probe. Using this approach, we also analyzed whether A4V, L38V, G93A, and G93C fALS mutations affected hSod1 function. We found that the levels of ROS observed in cells carrying an empty vector were similar to those observed in cells transfected with hSod1 constructs. Thus, we then asked whether we could detect differences in the handling of ROS in cells transfected with the hSod1 constructs. After 30 min of exposure of the cells expressing the different hSod1 variants to hydrogen peroxide (H_2_O_2_), we found that the WT, but not the mutant hSod1, reduced the levels of ROS (Figs. 5 and 6). This suggests that the fusions of WT hSod1 with the BiFC Venus protein fragments retain dismutase function, leading to reduced levels of ROS. In contrast, the Sod1 mutants lacked dismutase activity, suggesting the mutations affected the antioxidant activity of Sod1. This suggests the following: (i) the fusions of WT hSod1 with the BiFC Venus protein fragments do not impair the antioxidant role of the enzyme; (ii) A4V, L38V, G93A, and G93C fALS mutations cause hSod1 loss of function.

Next, to investigate whether the different hSod1 variants protected from DNA damage, we measured DNA single- and double-strand breaks using the comet assay. In this assay, the tail moment is expressed as the relation between the tail length (the smallest detectable size of migrating DNA) and the fraction of total DNA in the tail (the number of relaxed/broken pieces) [26]. Interestingly, we found that WT hSod1 protected against DNA damage, confirming that the BiFC tags did not affect Sod1 activity. In contrast, expression of A4V, L38V, and G93C hSod1 mutants did not protect against DNA damage as WT hSod1, having tail moments about five times larger, suggesting that these fALS Sod1 mutants are not able to maintain genomic stability due to their reduced presence in the nucleus (Fig. 1d). Strikingly, cells expressing the G93A hSod1 mutant exhibited the highest levels of DNA damage, which were about 50% higher than those observed for cells expressing WT Sod1.

## Discussion

hSod1 mutations associated with fALS are prone to misfolding and aggregation [27]. Various in vitro and in vivo studies have assessed the effects of fALS-associated mutations on hSod1, in an attempt to afford novel insight into the molecular basis of the disease [13–15, 17, 28–31]. Several studies suggested that the toxicity of hSod1 mutants is related with the respective amino acid substitution [32] and with alterations in the intracellular localization of the protein [11, 25, 33]. Moreover, the cytotoxic mechanisms exerted by Sod1 mutants have also been associated with a bulk saturation of clearance mechanisms (e.g., proteasomal or autophagic protein degradation), saturation of chaperone function and dysfunction of mitochondria, alterations in apoptotic pathways, and axonal disorganization and disrupted axonal transport [34–37]. However, the precise molecular underpinnings of fALS are still unclear.

The aggregation of mutant Sod1 in living cells and the alteration in conformation and localization have been monitored by tagging the protein with fluorescent fluorophores [31, 38]. Although such approaches may alter the biological function of proteins, they enable important studies that cannot be performed otherwise.

Given that hSod1 spontaneously dimerizes in normal conditions and forms a homodimer [39], we took advantage of the BiFC assay, based on the complementation of two non-fluorescent fragments of the fluorescent protein, in order to investigate the effect of fALS-associated mutants on hSod1 dimerization, oligomerization and aggregation, and on functional readouts of activity.

Initially, we observed that BiFC-tagged Sod1 A4V, L38V, G93A, and G93C dimerized and formed intracellular inclusions. This effect was particularly strong for the A4V mutant, which significantly increase in the percentage of cells with inclusions.

It is still unclear whether Sod1 inclusions are toxic, and additional studies using different model systems will be instrumental to address this. Recent studies have connected protein inclusions with SGs [24]. Using spinal cord motor neurons [40] or HeLa cells as models [23], it was shown that inclusions formed by G93A and A4V Sod1 mutants colocalize with G3BP, an established marker of SGs. In our study, we did not observe colocalization of the inclusions formed with G3BP, suggesting differences in the types of inclusions formed. Our observations are consistent with those reported recently, where inclusions of G93A Sod1 did not colocalize with SGs in HEK 293 cells [40]. Thus, our results suggest that, in normal conditions, the Sod1 mutants studied do not colocalize with SGs.

Sod1 aggregation is directly dependent of dimer dissociation, which is promoted by mutations that weaken the interactions at the dimer interface [41, 42]. FRET analysis indicated that fALS mutations impaired Sod1 dimerization, consequently affecting protein aggregation [17]. In addition, it was reported that mutant Sod1 tagged with YFP formed cytoplasmic inclusions in cultured cells, in contrast to WT Sod1 [43]. Consistently, WT Sod1-GFP chimeric protein maintain its dismutase activity and does not really form inclusions when expressed in PC12 cultured cells [44]. Thus, considering only the number of inclusions counted per cell, the A4V mutation promoted the most harmful effect in comparison to the other mutations. Live-cell imaging revealed that A4V and G93A mutations formed inclusion faster than G93C and L38V, suggesting, for the first time, that Sod1 variants A4V and G93A need the same time to aggregate and have more propensities to form inclusions over a time course. It has been shown that A4V variant had the most considerable dimerization defect and the strongest Sod1 aggregation activity, which is in agreement with the earlier aggregate formation observed in Fig. 3 [17]. In addition, G93A accumulated at lower levels, indicating that this altered protein is more rapidly degraded in cells. In comparison to other mutations related to fALS, G93A showed early disease onset related to the worst prognostic [45]. Moreover, in vitro studies that investigated the correlation between the propensity for aggregation and conformational stability of Sod1, showed that G93A have the highest conformation instability compared to other mutations, including A4V [46]. These fALS-linked mutations are located throughout the three-dimensional structure of the Sod1 protein. The G93A is located on turns of β-sheets of the β-barrel, and the A4V mutation is at the dimer interface. Studies using spectroscopic techniques [47, 48], thermal stability measurement [49], and disulfide reduction [32] have demonstrated that G93A and A4V mutation contribute to a reduction in protein stability, which may be related to their toxic gain of function. It has been shown that accumulation of toxic G93A Sod1 in the JUNQ compartment interferes with the quality control function, inhibiting the degradation of other misfolded proteins by sequestering Hsp70, thereby blocking proteasomal function. It was previously demonstrated that Sod1 A4V is a JUNQ-like aggregate which colocalizes with ubiquitin [33]. Thus, we verified whether the BiFC-tagged G93A Sod1 was indeed directed to JUNQ. Remarkably, we observed that all mutations analyzed colocalized with VHL protein. Thus, the mutant Sod1 localization in the JUNQ was confirmed a well-established hallmark feature of fALS, which increases the harmful effects of the mutations.

In addition to the observation of Sod1 inclusions, we observed that about 40% of dimeric Sod1 WT is located in the nucleus, while only 20% of mutant Sod1 was found in this compartment, showing a significant increase in fluorescence intensity of mutSod1 in the cytoplasm. These results indicate that the presence of Sod1 mutations decreases the ability to this protein to move into the nucleus. It has been shown in yeast cells that exposure to oxidants is sufficient to promote Sod1 nuclear localization through Dun1-Sod1 interaction and regulation of Sod1 by phosphorylation at S60, 99 [5]. In the nucleus, Sod1 affects the expression of oxidative resistance and repair genes [5] and interacts with DNA [50].

In this study, we confirmed that BiFC-tagged Sod1 mutants respond differently to mild exposure to H_2_O_2_ in comparison to WT Sod1. Sod1 mutants do not properly respond against intracellular ROS production. One possible explanation is that A4V, G93A, G93C, and L38V hSod1 as aggregates at cytoplasm are unable to enter the nucleus (Fig. 1d). Another possibility is that the fALS mutations impair the ROS signaling that mediates hSod1 nuclear translocation; by remaining at cytoplasm, fALS hSod1 mutants aggregate.

Although all fALS-associated hSod1 mutants showed a similar distribution between cytosol and nucleus (80 versus 20%), leading to an impaired ability to prevent DNA damage when compared to WT Sod1, G93A hSod1 exhibited the worst performance. The levels of damage were twofold higher in G93A hSod1 expressing cells than in cells which contain only the endogenous Sod1, empty vector, meaning that G93A hSod1 is non-active and harmful with respect to DNA protection.

Our study reveals an in vivo aggregation timeline between Sod1 mutants, which were probably responsible to induce DNA damage as well as a decreased nuclear localization in comparison to the WT form. Regarding the stages of ALS disease when Sod1 aggregates are formed, some aspects have been reported. Sod1 aggregate was found to accumulate at the highest levels as symptoms appear in mouse models of fALS [45, 51]. Moreover, inclusion-like structures were seen in symptomatic mice that express the ALS mutant G85R-SOD1, especially in mice motor neuron at the end-stage paralysis [52]. More recently, a timing of mutant Sod1 aggregation was reported in prion-like transmission studies with G85R-Sod1 expressing mice [53]. ALS patients harboring the A4V or G93A mutation have a short mean survival time of only about 1.5 years post diagnosis [17, 54]. On the contrary, G93C mutation patients present a long survival time [17]. The ephemeral survival time of fALS patients carrying G93A or A4V mutations correlates with the fastest aggregation observed in cells in the current study. Ultimately, our findings may impact on the development of therapeutic strategies aimed at modulating the aggregation of Sod1, by providing novel and detailed information about the behavior of WT and mutant Sod1 in living cells.

## References

[1] Rowland LP, Shneider NA (2001). Amyotrophic lateral sclerosis. N Engl J Med.

[2] Schymick JC, Talbot K, Traynor BJ (2007). Genetics of sporadic amyotrophic lateral sclerosis. Hum Mol Genet.

[3] Abe K, Aoki M, Ikeda M (1996). Clinical characteristics of familial amyotrophic lateral sclerosis with Cu/Zn superoxide dismutase gene mutations. J Neurol Sci.

[4] Fukai T, Ushio-Fukai M (2011). Superoxide dismutases: role in redox signaling, vascular function, and diseases. Antioxid Redox Signal.

[5] Tsang CK, Liu Y, Thomas J et al (2014) Superoxide dismutase 1 acts as a nuclear transcription factor to regulate oxidative stress resistance. Nat Commun. 10.1038/ncomms4446 10.1038/ncomms4446 PMC4678626 24647101

[6] Valentine JS, Doucette PA, Zittin Potter S (2005). Copper-zinc superoxide dismutase and amyotrophic lateral sclerosis. Annu Rev Biochem.

[7] Valdmanis PN, Daoud H, Dion PA, Rouleau GA (2009). Recent advances in the genetics of amyotrophic lateral sclerosis. Curr Neurol Neurosci Rep.

[8] Matsumoto G, Stojanovic A, Holmberg CI (2005). Structural properties and neuronal toxicity of amyotrophic lateral sclerosis-associated Cu/Zn superoxide dismutase 1 aggregates. J Cell Biol.

[9] Bruijn LI, Miller TM, Cleveland DW (2004). Unraveling the mechanisms involved in motor neuron degeneration in ALS. Annu Rev Neurosci.

[10] Allen S, Badarau A, Dennison C (2012). Cu (I) affinities of the domain 1 and 3 sites in the human metallochaperone for Cu,Zn-superoxide dismutase. Biochemistry.

[11] Weisberg SJ, Lyakhovetsky R, Werdiger AC (2012). Compartmentalization of superoxide dismutase 1 (SOD1G93A) aggregates determines their toxicity. Proc Natl Acad Sci.

[12] Tu PH, Raju P, Robinson K a (1996). Transgenic mice carrying a human mutant superoxide dismutase transgene develop neuronal cytoskeletal pathology resembling human amyotrophic lateral sclerosis lesions. Proc Natl Acad Sci.

[13] Roberts K, Zeineddine R, Corcoran L (2013). Extracellular aggregated Cu/Zn superoxide dismutase activates microglia to give a cytotoxic phenotype. Glia.

[14] Yerbury JJ, Gower D, Vanags L (2013). The small heat shock proteins αb-crystallin and Hsp27 suppress SOD1 aggregation in vitro. Cell Stress Chaperones.

[15] Furukawa Y, Kaneko K, Yamanaka K (2008). Complete loss of post-translational modifications triggers fibrillar aggregation of SOD1 in the familial form of amyotrophic lateral sclerosis. J Biol Chem.

[16] McAlary L, Aquilina JA, Yerbury JJ (2016). Susceptibility of mutant SOD1 to form a destabilized monomer predicts cellular aggregation and toxicity but not in vitro aggregation propensity. Front Neurosci.

[17] Kim J, Lee H, Lee JH (2014). Dimerization, oligomerization, and aggregation of human amyotrophic lateral sclerosis copper/zinc superoxide dismutase 1 protein mutant forms in live cells. J Biol Chem.

[18] Gralla EB, Valentine JS (1991). Null mutants of *Saccharomyces cerevisiae* Cu,Zn superoxide dismutase: characterization and spontaneous mutation rates. J Bacteriol.

[19] Brasil AA, Belati A, Mannarino SC (2013). The involvement of GSH in the activation of human Sod1 linked to FALS in chronologically aged yeast cells. FEMS Yeast Res.

[20] Outeiro TF, Putcha P, Tetzlaff JE et al (2008) Formation of toxic oligomeric α-synuclein species in living cells. PLoS One. 10.1371/journal.pone.0001867 10.1371/journal.pone.0001867 PMC2270899 18382657

[21] Olive PL, Banáth JP (2006). The comet assay: a method to measure DNA damage in individual cells. Nat Protoc.

[22] Gonçalves SA, Matos JE, Outeiro TF (2010). Zooming into protein oligomerization in neurodegeneration using BiFC. Trends Biochem Sci.

[23] Mateju D, Franzmann TM, Patel A (2017). An aberrant phase transition of stress granules triggered by misfolded protein and prevented by chaperone function. EMBO J.

[24] Siwach P, Kaganovich D (2017). Getting stress out of stressed‐out stress granules. EMBO J.

[25] Kaganovich D, Kopito R, Frydman J (2008). Misfolded proteins partition between two distinct quality control compartments. Nature.

[26] Fairbairn DW, Olive PL, O’Neill KL (1995). The comet assay: a comprehensive review. Mutat Res Genet Toxicol.

[27] Kaur SJ, McKeown SR, Rashid S (2016). Mutant SOD1 mediated pathogenesis of amyotrophic lateral sclerosis. Gene.

[28] Park J-H, Park H-S, Hong S, Kang S (2016). Motor neurons derived from ALS-related mouse iPS cells recapitulate pathological features of ALS. Exp Mol Med.

[29] Wei R, Bhattacharya A, Hamilton RT (2013). Differential effects of mutant SOD1 on protein structure of skeletal muscle and spinal cord of familial amyotrophic lateral sclerosis: role of chaperone network. Biochem Biophys Res Commun.

[30] Watanabe M, Dykes-Hoberg M, Culotta VC (2001). Histological evidence of protein aggregation in mutant SOD1 transgenic mice and in amyotrophic lateral sclerosis neural tissues. Neurobiol Dis.

[31] Ayers JI, McMahon B, Gill S et al (2016) Relationship between mutant SOD1 maturation and inclusion formation in cell models. J Neurochem. 10.1111/jnc.13864 10.1111/jnc.13864 PMC5283795 27727458

[32] Tiwari A, Hayward LJ (2003). Familial amyotrophic lateral sclerosis mutants of copper/zinc superoxide dismutase are susceptible to disulfide reduction. J Biol Chem.

[33] Farrawell NE, Lambert-Smith IA, Warraich ST (2015). Distinct partitioning of ALS associated TDP-43, FUS and SOD1 mutants into cellular inclusions. Sci Rep.

[34] Taylor JP, Brown RH, Cleveland DW (2016). Decoding ALS: from genes to mechanism. Nature.

[35] Tafuri F, Ronchi D, Magri F et al (2015) SOD1 misplacing and mitochondrial dysfunction in amyotrophic lateral sclerosis pathogenesis. Front Cell Neurosci. 10.3389/fncel.2015.00336 10.3389/fncel.2015.00336 PMC4548205 26379505

[36] Keskin I, Forsgren E, Lange DJ (2016). Effects of cellular pathway disturbances on misfolded superoxide dismutase-1 in fibroblasts derived from ALS patients. PLoS One.

[37] Tiwari A, Hayward LJ (2006). Mutant SOD1 instability: implications for toxicity in amyotrophic lateral sclerosis. Neurodegener Dis.

[38] Zhang F, Zhu H (2006). Intracellular conformational alterations of mutant SOD1 and the implications for fALS-associated SOD1 mutant induced motor neuron cell death. Biochim Biophys Acta Gen Subj.

[39] Saccon RA, Bunton-Stasyshyn RKA, EMC F, Fratta P (2013). Is SOD1 loss of function involved in amyotrophic lateral sclerosis?. Brain.

[40] Gal J, Kuang L, Barnett KR (2016). ALS mutant SOD1 interacts with G3BP1 and affects stress granule dynamics. Acta Neuropathol.

[41] Broom HR, Rumfeldt JAO, Vassall KA, Meiering EM (2015). Destabilization of the dimer interface is a common consequence of diverse ALS-associated mutations in metal free SOD1. Protein Sci.

[42] Fay JM, Zhu C, Proctor EA (2016). A phosphomimetic mutation stabilizes SOD1 and rescues cell viability in the context of an ALS-associated mutation. Structure.

[43] Prudencio M, Durazo A, Whitelegge JP, Borchelt DR (2010). An examination of wild-type SOD1 in modulating the toxicity and aggregation of ALS-associated mutant SOD1. Hum Mol Genet.

[44] Stevens JC, Chia R, Hendriks WT et al (2010) Modification of superoxide dismutase 1 (SOD1) properties by a GFP tag—implications for research into amyotrophic lateral sclerosis (ALS). PLoS One. 10.1371/journal.pone.0009541 10.1371/journal.pone.0009541 PMC2833207 20221404

[45] Karch CM, Prudencio M, Winkler DD (2009). Role of mutant SOD1 disulfide oxidation and aggregation in the pathogenesis of familial ALS. Proc Natl Acad Sci U S A.

[46] Stathopulos PB, Rumfeldt JAO, Scholz GA (2003). Cu/Zn superoxide dismutase mutants associated with amyotrophic lateral sclerosis show enhanced formation of aggregates in vitro. Proc Natl Acad Sci U S A.

[47] Liu H, Zhu H, Eggers DK (2000). Copper (2+) binding to the surface residue cysteine 111 of His46Arg human copper-zinc superoxide dismutase, a familial amyotrophic lateral sclerosis mutant. Biochemistry.

[48] Shipp EL, Cantini F, Bertini I (2003). Dynamic properties of the G93A mutant of copper-zinc superoxide dismutase as detected by NMR spectroscopy: Implications for the pathology of familial amyotrophic lateral sclerosis. Biochemistry.

[49] Hayward LJ, Rodriguez JA, Kim JW (2002). Decreased metallation and activity in subsets of mutant superoxide dismutases associated with familial amyotrophic lateral sclerosis. J Biol Chem.

[50] Barbosa LF, Cerqueira FM, Macedo AFA (2010). Increased SOD1 association with chromatin, DNA damage, p53 activation, and apoptosis in a cellular model of SOD1-linked ALS. Biochim Biophys Acta Mol Basis Dis.

[51] Wang J, Xu G, Li H (2005). Somatodendritic accumulation of misfolded SOD1-L126Z in motor neurons mediates degeneration: αB-crystallin modulates aggregation. Hum Mol Genet.

[52] Wang J, Farr GW, Zeiss CJ (2009). Progressive aggregation despite chaperone associations of a mutant SOD1-YFP in transgenic mice that develop ALS. Proc Natl Acad Sci U S A.

[53] Ekhtiari Bidhendi E, Bergh J, Zetterström P (2016). Two superoxide dismutase prion strains transmit amyotrophic lateral sclerosis-like disease. J Clin Invest.

[54] Galaleldeen A, Strange RW, Whitson LJ (2009). Structural and biophysical properties of metal-free pathogenic SOD1 mutants A4V and G93A. Arch Biochem Biophys.

